# A novel approach for the discovery of chemically diverse anti-malarial compounds targeting the *Plasmodium falciparum* Coenzyme A synthesis pathway

**DOI:** 10.1186/1475-2875-13-343

**Published:** 2014-08-31

**Authors:** Sabine Fletcher, Vicky M Avery

**Affiliations:** Discovery Biology, Eskitis Institute for Drug Discovery, Griffith University, Nathan, Queensland, 4111 Australia

**Keywords:** Malaria, Medicines for malaria venture (MMV) malaria box, *Plasmodium falciparum*, Coenzyme A synthesis, Anti-malarial compounds, HTS drug discovery

## Abstract

**Background:**

Malaria is a devastating parasitic disease, causing more than 600,000 deaths annually. Drug resistance has rendered previous generation anti-malarials ineffective and is also rapidly emerging against the current therapeutics of choice, artemisinin and its derivatives, making the discovery of new anti-malarials with novel mechanisms of action a priority. The Coenzyme A (CoA) synthesis pathway, a well-known anti-microbial drug target that is also essential for the malaria parasite *Plasmodium falciparum*, has not yet been exploited in anti-malarial drug development. A novel high throughput approach for the identification of chemically diverse inhibitors of the CoA synthesis pathway is reported.

**Methods:**

To identify novel CoA synthesis pathway inhibitors, a chemical rescue screening approach was developed. In short, a test compound was considered likely to inhibit the *P. falciparum* CoA synthesis pathway, if addition of the end product of the pathway, CoA, was able to negate the growth-inhibitory action of the compound on *P. falciparum* parasites.

**Results:**

The chemical rescue approach was employed to screen the Medicines for Malaria Venture malaria box and a small focussed compound library. This resulted in the identification of 12 chemically diverse potential inhibitors of the CoA pathway. To ascertain accurate potency and selectivity, the half-maximal inhibitory concentration (IC_50_ value) of these compounds was determined for both *P. falciparum* and a human cell line. Seven compounds showed submicromolar activity against the parasite, with selectivity indices ranging between six and greater than 300. CoA supplementation was confirmed to alleviate the effects on parasite growth and cell viability in a dose dependent manner. Microscopic investigation into the stage of effect and phenotype of treated parasites was performed on a selection of the active compounds.

**Conclusions:**

The chemical rescue approach described resulted in the identification of a set of chemically diverse CoA synthesis pathway inhibitors with IC_50_ values ranging between 120 nM and 6 μM. The identified compounds will be utilized as tools for further investigating the parasite CoA synthesis pathway to define their exact mechanism of action. Furthermore, the chemical diversity of the compounds identified substantiates the suitability of this approach to identify novel starting points for future anti-malarial drug development.

**Electronic supplementary material:**

The online version of this article (doi:10.1186/1475-2875-13-343) contains supplementary material, which is available to authorized users.

## Background

Despite decades of concerted efforts undertaken to control malaria, this tropical infectious disease still causes hundreds of millions of clinical episodes each year, of which over 600,000 end fatally [1]. The majority of deaths can be attributed to *Plasmodium falciparum* and the efficacy of currently used drugs is jeopardized by the emergence of drug-resistant strains of this parasite [2]. Development of widespread resistance has already led to significantly decreased efficiency of traditional anti-malarial drugs, such as chloroquine and pyrimethamine [2]. Furthermore, the development of resistance against the present generation drug, artemisinin and its derivatives, has also been observed [2, 3]. This clearly demonstrates the need for anti-malarial drugs with novel mechanisms of action and/or of different chemical origin to effectively counteract the development of resistance, thus reinforcing the current defense against malaria [3, 4].

**Figure 1:**
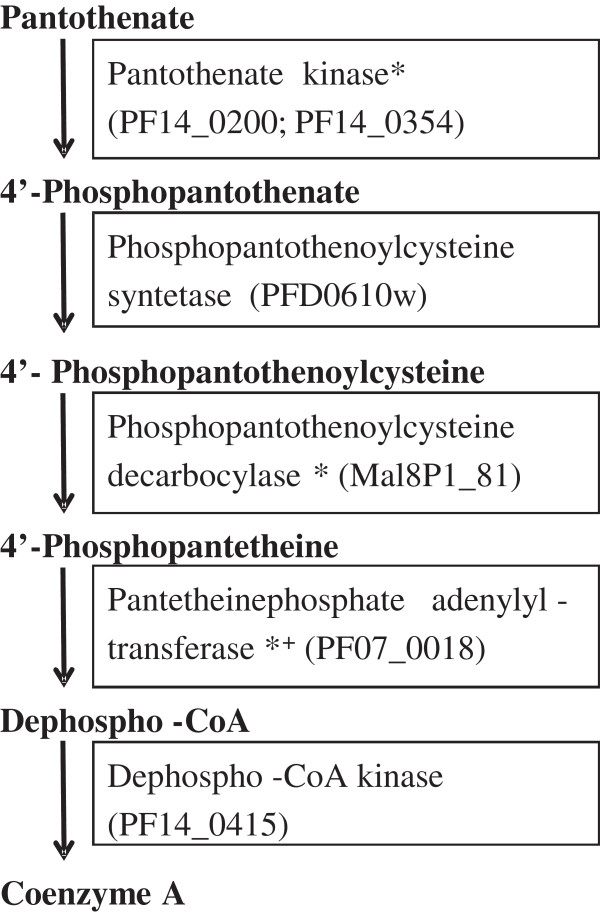
**Diagram of the coenzyme A synthesis pathway.** The five enzymatic steps of CoA synthesis are highly conserved, however the amino acid sequences of the enzymes catalysing the reactions show low conservation between species. Identifiers of the putative *P. falciparum* enzymes are shown in brackets; * Essentiality of these putative *P. falciparum* enzymes is predicted in [6]; # Essentiality is predicted in [7].

Novel targets suitable for rational drug discovery need to fulfil certain requirements. Firstly they should be essential for parasite survival to avoid low-level survival due to redundant processes, which in turn could facilitate development of drug resistance. The *P. falciparum* genome contains putative enzymes for all five steps of Coenzyme A (CoA) synthesis [5] (Figure 1). Several of these enzymes have been predicted to be essential for the malaria parasite by means of *in silico* metabolomic investigations [6, 7] (Figure 1). Importantly, *P. falciparum* survival was shown to be independent of host CoA biosynthesis, indicating a distinct capability of de-novo CoA synthesis [8]. Furthermore, pro-vitamin B5 (panthenol), as well as several analogues, have previously been demonstrated to inhibit the *in vitro* growth of *P. falciparum* [9, 10]. Collectively, these facts suggest that the CoA synthesis pathway is indeed essential for parasite survival.

In addition to being essential in *P. falciparum*, a novel drug target should be either unique to the parasite or sufficiently divergent from the host, to avoid unwanted interference with the host metabolism, e.g., toxicity. Despite the conservation of the five enzymatic steps involved in CoA synthesis in eukaryotes [11], the amino acid sequence of the enzymes involved are not highly conserved between species [7]. For pantothenate kinase, the enzyme catalysing the first and rate-limiting step of CoA synthesis [11], the maximal amino acid sequence homology between the known human isotypes and the two putative *P. falciparum* proteins PF14_0354 and PF14_0200 as retrieved from PlasmoDB [12], is only 26-33% determined by BLAST analysis [13]. Therefore, the enzymes of the human and parasite CoA pathway are expected to be divergent enough to allow the identification of compounds that act selectively on *P. falciparum*.

This study reports the use of a novel chemical rescue approach to identify chemically diverse inhibitors of the *P. falciparum* CoA synthesis pathway from a set of prioritised compounds, as well as the Medicines for Malaria Venture (MMV) malaria box, both with known asexual activities. The antiplasmodial potency and parasite specificity of the identified inhibitors was determined in dose response. The observed chemical rescue of parasite growth and cell viability was shown to be a dose-dependent effect. Investigations into the specific parasite stage affected by the compounds and phenotypic changes in treated parasites are reported for seven submicromolar active compounds.

## Methods

### *Plasmodium falciparum*culture

*In vitro* parasite culture was maintained in RPMI with 10 mM Hepes (Life Technologies), 50 μg/ml hypoxanthine (Sigma) and 5 mg/ml AlbuMAX II® (Life Technologies). Human O + erythrocytes were obtained from the Australian Red Cross Blood Service (Agreement No: 13-04QLD-09). The parasites were maintained at 2-8% parasitaemia (% P) at 5% haematocrit (% H), incubated at 37°C, 5% CO_2_, 5%O_2_ and 90% N_2_ and 95% humidity.

### *Plasmodium falciparum*growth inhibition and Coenzyme A chemical rescue assay

The well-established *P. falciparum* imaging assay [14] was modified to allow assessment of chemical rescue with CoA. In brief, sorbitol (5% w/v) synchronization was performed twice, approximately 8 hr apart, on each synchronization day for two consecutive ring cycles, i.e., on day 1 and 3 of assay preparation. On day 2 the culture was split to approximately 2% trophozoite parasitaemia. On day 4 the culture was split to 1-1.5% trophozoite parasitaemia, which yielded approximately 8% ring parasitaemia after 48 hr (on day 5, i.e., the day of the assay set-up).

Compound stocks (10 mM in 100% DMSO) were diluted 1:25 in sterile water, less than 24 hr prior to use. An additional 1:10 dilution was performed, resulting in a 1:250 overall compound dilution and a final DMSO concentration of 0.4%. For initial screening, compounds were tested at a final 2 μM concentration. For dose response curves of the active compounds, a semi-logarithmic serial dilution (containing three data points per log) was prepared at 40 μM top concentration for test compounds and 2 μM for the positive control, artemisinin. The known *P. falciparum* CoA synthesis inhibitor, panthenol, was prepared as a 100 mM solution in 4% DMSO. The final concentration of the dose response curve of panthenol started at 10 mM and was serially diluted down to 100 nM. CoA was dissolved in water. The final CoA concentration for optimal rescue results was determined for each CoA batch and ranged between 0.8-2 mM.

Five μl of the 1:25 diluted test compound or control solutions were added to 384-well CellCarrier imaging plates (PerkinElmer). For the chemical rescue, 5 μl of CoA solution were added to ‘rescue’ wells and 5 μl water to all control and reference wells, respectively. Parasite culture was added to a final concentration of 1% parasitaemia (% P) and 0.3% haematocrit (% H). Plates were incubated for 72 hr at 37°C, 5% CO_2_ and 95% humidity. On day 8 the permeabilization and nuclear staining buffer was prepared in PBS containing 10 μg/ml saponin, 0.01% triton X, 5 mM EDTA (all: Sigma) and 0.5 μg/ml 4′,6-diamidino-2-phenylindole (DAPI; Life Technologies) [14].

The plates were left for at least 4 hr (typically overnight), before confocal imaging on an Opera™ High Content Screening Platform (PerkinElmer) at 405 nm excitation with a 20x water objective. Automated primary image analysis was performed concurrent with the imaging process, utilizing an Acapella® software (PerkinElmer) script to determine the number of parasites based on object size and fluorescence intensity [14]. Determination of the % growth compared to controls (2 μM artemisinin as positive and 0.4% DMSO as negative control) was performed in Microsoft® Excel 2010. Statistical analysis including IC_50_ determination and graphical output was performed in GraphPad Prism® 5 using non-linear regression variable slope curve fitting.

### HEK293 viability assay

To assess cytotoxicity of compounds in dose response, a resazurin-based assay was utilized to determine cell viability. In brief, HEK293 cells were grown in DMEM medium (Life Technologies) containing 10% foetal calf serum (FCS; Gibco). Cells were trypsinized, counted and seeded at 2,000 cells per well in 45 μl media into TC-treated 384-well plates (Falcon) and left to adhere overnight at 37°C, 5% CO_2_ and 95% humidity.

Test compounds were prepared as described above, to give a top final test concentration of 40 μM, 0.4% DMSO. Plates were incubated for 72 hr at 37°C, 5% CO_2_ and 95% humidity, then the media was removed and replaced by 35 μl of 44 μM resazurin in DMEM without FCS. The plates were incubated for another 4-6 hr at 37°C, 5% CO_2_ and 95% humidity before reading on an EnVision® Plate Reader (PerkinElmer) using fluorescence excitation/emission settings of 530 nm/595 nm. Determination of the% growth compared to controls (40 μM puromycin as positive and 0.4% DMSO as negative control) was performed in Microsoft® Excel 2010. Statistical analysis including IC_50_ determination and graphical output was performed in GraphPad Prism® 5 using nonlinear regression variable slope curve fitting.

### Time course for assessment of parasite stage affected and phenotype of treated parasites

The principle of the *P. falciparum* imaging assay described above was used, with the exception that the parasite synchronization was adjusted to yield parasites synchronized to a 2-hr window as established in-house. In brief, sorbitol synchronization was performed twice (approximately 8 hr apart) on day 1 and only once on day 3 (around 10.00). Schizonts observed on day 4 were left to mature and when rings were first observed, the mature schizonts were isolated using a MACS® column (Miltenyi Biotec). The purified schizonts were incubated for 2 hr at 37°C, 5% CO_2_ and 5% O_2_ and 90% N_2_ on a shaker. The obtained 0-2 hr old ring stage parasites were isolated by removing the remaining schizonts by flow through a MACS® column and the highly synchronized rings obtained were seeded in the assay plates with the compounds as described above. At each time point, one whole columns (16 wells) of identically treated parasites, i.e., 0.4% DMSO as control, compound at its individual IC_80_ or compound at IC_80_ plus CoA, were pooled and centrifuged. A smear was made from the pellet and stained with Giemsa (Sigma) for assessment the phenotype of treated parasites with or without supplementation of 0.8 mM CoA in comparison to DMSO controls.

## Results

### *Plasmodium falciparum*growth inhibitory action of panthenol is exclusively due to inhibition of the Coenzyme A synthesis pathway

**Figure 2:**
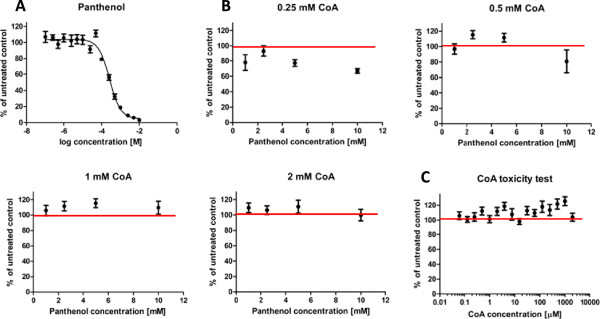
**Coenzyme A rescues panthenol-induced** ***Plasmodium falciparum*** **growth inhibition. A**: Confirmation of panthenol as a dose-dependent *P. falciparum* growth inhibitor; graphs represent n = 3, triplicate point each; error bars indicate +/- standard error of mean (SEM). **B**: Supplementation of CoA concentrations of 1 and 2 mM rescued panthenol-induced growth inhibition; graphs represent n = 3, triplicate point each; error bars indicate +/- standard error of mean (SEM). **C**: Parasite growth was not significantly altered by supplementation of CoA alone; graphs represent n = 3, triplicate point each; error bars indicate +/- standard error of mean (SEM).Different batches of CoA purchased throughout the course of this study varied in their ability to rescue the parasites, as well as varying in degrees of toxicity at higher concentrations. As a consequence, and to ensure comparable results were obtained, the CoA concentration used in each experiment was adjusted to an optimal rescue concentration for each CoA batch. The optimal rescue concentration varied from 0.8 to 2 mM.

Panthenol (Pro-vitamin B5) has been previously described as an inhibitor of *P. falciparum* growth [9]. Its action was due to the inhibition of pantothenate phosphorylation, the first step of the CoA synthesis pathway, which is catalysed by the enzyme pantothenate kinase [9]. The activity of panthenol on *P. falciparum* strain 3D7 was independently assessed using an established image-based growth inhibition assay [14, 15]. Panthenol was confirmed to function as a dose-dependent *P. falciparum* growth inhibitor (Figure 2A). To investigate whether the observed growth inhibition was solely due to interference with CoA synthesis, the growth medium was supplemented with increasing amounts of CoA (ranging from 60 nM to 2 mM final concentration) during treatment with panthenol. The growth inhibitory effect of panthenol could be fully rescued by supplementation of CoA at concentrations between 1 and 2 mM, demonstrating that CoA supplementation alone was sufficient to enable parasite survival under panthenol treatment (Figure 2B). To assess reproducibility, the Z-factor value was calculated both for the assay controls and the CoA rescue of the positive control panthenol from 3 independent experiments. For the assay controls of 100% growth inhibition (2 μM artemisinin) and no growth inhibition (0.4% DMSO) the Z-factor value ranged between 0.60 and 0.85, with a mean of 0.73 and a standard deviation of 0.1. With regard to the activity of 10 mM Panthenol, as well as its complete CoA rescue achieved with 1 mM and 2 mM CoA supplementation, the following Z-factor values were obtained: when rescued with 2 mM CoA, the Z’ values ranged between 0.66 and 0.72 with a mean of 0.70 and a standard deviation of 0.03; for the rescue with 1 mM CoA the Z’ ranged between 0.68 and 0.72, with an average value of 0.7 +/- 0.02 standard deviation. An assay with a Z-factor value between 1 and 0.5 is considered an excellent assay related to high throughput screening capability [16]. Parasite growth was not significantly altered by supplementation of CoA alone (Figure 2C). However, it is important to note that batch-specific variations in CoA toxicity were observed and as a consequence, the optimal rescue concentration was tested for each new CoA batch before use. Collectively, these results confirmed that the antiplasmodial action of panthenol is primarily due to inhibition of the parasite CoA synthesis pathway.

### Discovery of novel *Plasmodium falciparum*Coenzyme A synthesis inhibitors

Cell-based high throughput screening (HTS) entails the distinct benefit that positive hits have already proven effective against the live parasite within its natural host erythrocyte environment [17, 18]. Modifications of an image-based assay [14] were undertaken to identify compounds that interfere with the *P. falciparum* CoA pathway. The modification involved a chemical rescue approach adapted from a similar method used to investigate apicoplast function [19]. The principle idea of the chemical rescue approach is that supplementation of an unbranched pathway’s end product – in this case CoA - should abrogate, or at least alleviate, negative growth effects of compounds inhibiting any step in the pathway. In this assay, a test compound was considered likely to inhibit the CoA synthesis pathway of the parasite, if addition of CoA was able to negate the compound’s growth-inhibitory action. To identify new potential CoA synthesis inhibitors, the CoA chemical rescue approach was used to screen 144 compounds with known antiplasmodial activity identified by HTS in the Avery laboratory, in addition to the MMV malaria box [20]. Initially compound activity was directly compared at 2 μM with and without supplementation of CoA. The 2 μM compound concentration was deliberately chosen as an activity threshold, since compounds with an IC_50_ value above 2 μM are unlikely to progress further into drug lead development. In the CoA rescue assay, compounds were defined as hits if CoA supplementation could rescue the relative parasite number to ≥80% of untreated control levels with at least three-fold difference in relative parasite numbers between rescued and compound-treated samples. Compounds which rescued growth to at least 50% of control values and two-fold difference compared to treatment alone, were regarded as partial rescue hits.

Screening of the 144 compounds was performed in duplicate point for two biological replicates (n = 2), and yielded four hits and ten partial rescue hits. Screening of the MMV malaria box was initially conducted only in single dose and n = 1, due to the compound number and sample availability. A smaller subset of 31 compounds from the MMV malaria box, for which CoA supplementation demonstrated at least partial rescue, was retested in three biological replicates (n = 3). Of these, one compound (MMV665820) confirmed as a hit and three more as partial rescue hits under the abovementioned selection criteria.

**Figure 3:**
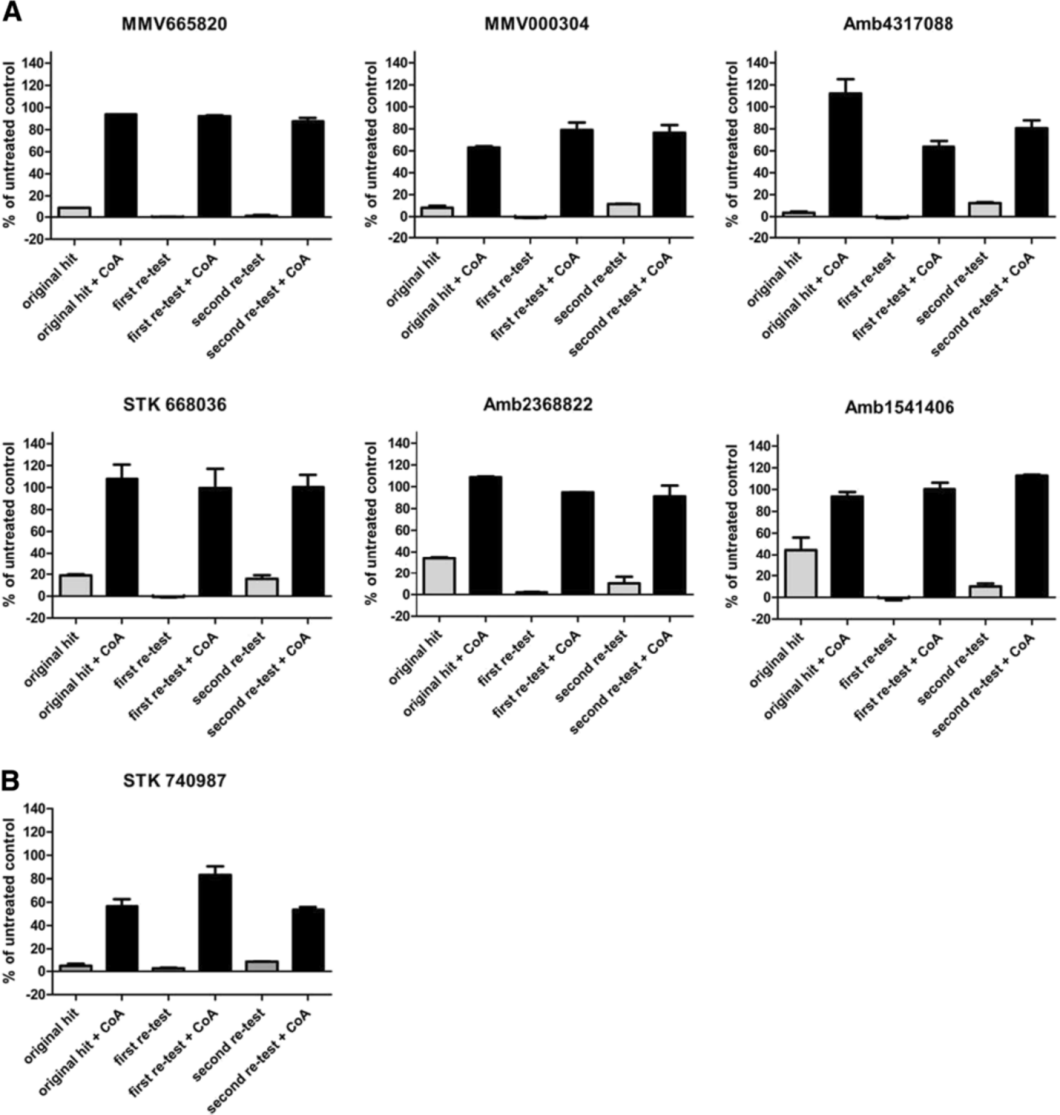
**Compound activity at 2 μM with and without supplementation of Coenzyme A. A**: Confirmed hits at 2 μM compound concentration. Direct comparison of compound activity with (black) and without (grey) addition of CoA. Shown are the original hits from compound library plates and two re-tests from purchased solids. Four compounds showed rescue levels of over 80% of untreated control values and two more of just below 80% when CoA was added; original hit bar graphs show n = 2, duplicate point for hits originating from the focussed compound library and n = 1, single point for compounds of the MMV malaria box. Re-test bar graphs show n = 1; duplicate point each; error bars indicate +/- standard error of mean (SEM). **B**: Confirmed hit with rescue to >50% of untreated control value at a 2 μM compound concentration. The original hit bar graph shows n = 2, duplicate point. Both re-test bar graphs show n = 1; duplicate point each; error bars indicate +/- standard error of mean (SEM).

All five of the identified hit compounds showing ≥80% rescue, as well as nine of the ≥50% rescue compounds, were obtained as solids and retested to confirm activity and CoA rescue responsiveness. Of the purchased compounds, four showed rescue levels greater than 80% and two approximately 80% at 2 μM compound concentration (Figure 3A). Of the remaining eight compounds, only one reconfirmed with greater than 50% rescue levels at a 2 μM concentration (Figure 3B).

### Evaluation of compound IC_50_and selectivity against *Plasmodium falciparum*

**Figure 4:**
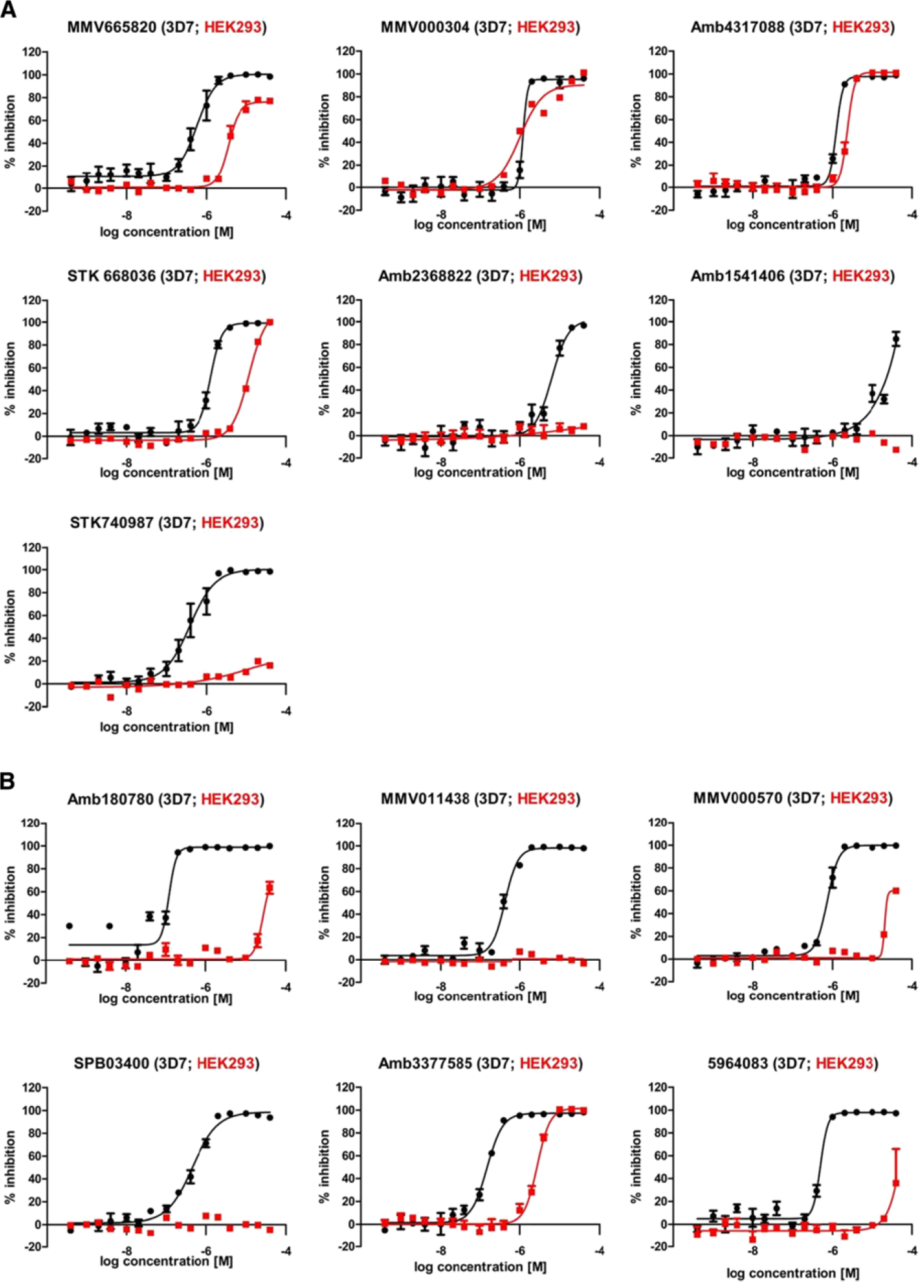
**Compound IC**_**50**_ **curves determined for** ***Plasmodium falciparum*** **strain 3D7 and human HEK293 cells. A**: IC_50_ curves of hits that confirmed in the CoA rescue at 2 μM compound concentration. IC_50_ curves against *P. falciparum* 3D7 are shown in black, against HEK293 in red; graphs depict n = 3 in duplicate or triplicate point each; error bars indicate +/- standard error of mean (SEM). **B**: IC_50_ curves of hits that did not confirm in the CoA rescue at 2 μM compound concentration. It was observed that these were generally the more potent antiplasmodial compounds. IC_50_ curves against *P. falciparum* 3D7 are shown in black, against HEK293 in red. Curves show n = 3 in duplicate or triplicate point each; error bars indicate +/- standard error of mean (SEM).

Accurate IC_50_ values were ascertained for all compounds, in addition to determining cytotoxicity against human embryonic kidney cells (HEK293) to demonstrate selectivity (Figure 4 and Table 1).

**Table 1:** **Overview of compound IC**_**50**_ **against** ***Plasmodium falciparum*** **and HEK293 cells**

	Structure	Compound ID	IC50 [μM]			IC50 [μM]			SI
			(3D7; n = 3)			(HEK293; n = 3)			
			Mean	+ SEM	- SEM	Mean	+ SEM	- SEM	
80% rescue at 2 μM		MMV665820/5534045	0.58	0.16	0.13	3.6	0.47	0.42	6.2
		MMV000304/Amb636705	1.21	0.30	0.24	1.00	0.33	0.25	0.8
		Amb4317088	1.23	0.12	0.11	2.33	0.24	0.22	1.9
		STK 668036	1.30	0.16	0.14	11.92	2.59	2.13	9.2
		Amb2368822/(MMV665980)	6.31	2.08	1.56	> 40			> 6.3
		Amb1541406	85% inhibition at 40 μM			> 40			ND
50% rescue at 2 μM		STK 740987	0.38	0.11	0.09	> 40			> 105
80% rescue at ~ IC_80_		Amb180780	0.12	0.03	0.02	~ 40			~ 333
		MMV011438/STK039514	0.43	0.05	0.05	> 40			> 93
		MMV000570/C614-0191	0.74	0.08	0.07	~ 40			~ 54
50% rescue at ~ IC_80_		SPB03400	0.45	0.09	0.07	> 40			89
		Amb3377585	0.15	0.02	0.02	2.75	0.36	0.32	18
Not con-firmed		5964083	0.50	0.09	0.08	> 40			> 80

The selectivity index (**SI**) of the compounds was calculated by dividing the HEK293 IC_50_ value by the *P. falciparum* IC_50_ value.

**SEM:** standard error of mean.

**ND:** not determined, since no IC_50_ values could be obtained.

Eight compounds showed no or only minor cytotoxicity against human HEK293 cells at the highest concentration tested (40 μM). This resulted in high selectivity indices (SI) of 50 to >300 times for six of the compounds. Five compounds had moderate selectivity indices between 5 and 18. Only two compounds (Amb4317088 and MMV000304) showed no parasite specificity and one compound was found to be of too low activity against *P. falciparum* to calculate a SI (Figure 4 and Table 1).

The relatively high IC_50_ value of 6.3 μM obtained for compound Amb2368822 was unexpected, since the deliberately chosen activity cut-off was greater than 50% activity at 2 μM. In actual fact, the compound had shown 66% parasite growth inhibition at a 2 μM concentration in the original single dose screen (original hit from focussed compound library plate). When retested twice from freshly dissolved solid, this compound displayed 98 and 90% inhibition at 2 μM (Figure 3A). The significantly lower activity observed during IC_50_ value generation from the same stock solution at a later time suggests that the compound is potentially unstable, irrespective of the storage conditions used. Standard practice is to store compounds in 100% DMSO at -20°C and minimal freeze-thaw cycles (1-2). Interestingly, the same compound was also present in the MMV malaria box (MMV665980), however it was not identified as a hit when screening the MMV malaria box, since here it showed only 27% inhibition at 2 μM. This indicates again a reduced antiplasmodial activity at time of testing, likely associated with compound degradation/instability or light sensitivity.

### Re-evaluation of higher potency compounds at IC_80_

**Figure 5:**
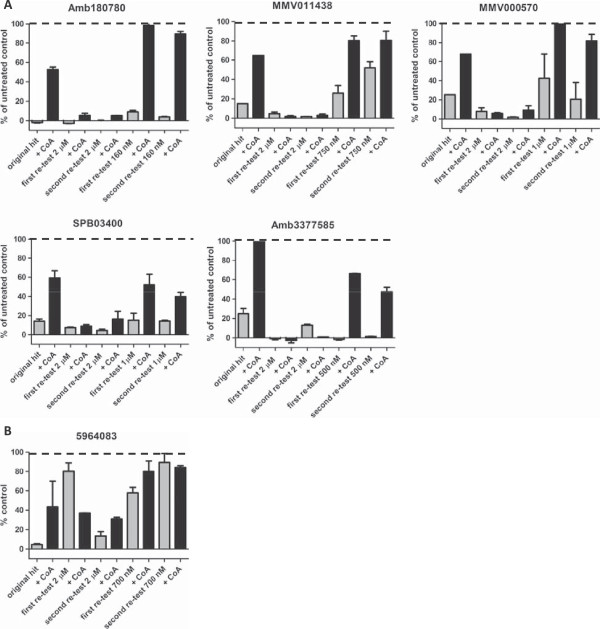
**Re-evaluation of higher potency compounds near individual IC**_**80**_ **. A**: Compounds that did not confirm at 2 μM concentration underwent additional testing at a lower concentration near their individual IC_80_ values. Direct comparison of compound activity with (black) and without (grey) addition of CoA is shown. At the indicated concentrations, three compounds were rescued to levels of ≥80% (top row) and two to approximately 50% of untreated control values (bottom row). Dashed lines indicate 100% of untreated control values, bars reaching values over 100% are shown as 100% . The bar graphs for original hits show n = 2, duplicate point for hits originating from the focussed compound library and n = 1, single point for compounds of the MMV malaria box. Re-test bar graphs show n = 1; duplicate point each; error bars indicate +/- standard error of mean (SEM). **B**: Compound 5964083 was the only compound that failed to reconfirm both at 2 μM and at a concentration near its IC_80_ value. The bar graph of the original hit depicts n = 2, duplicate point. All re-test bar graphs show n = 1; duplicate point each; error bars indicate +/- standard error of mean (SEM).

Comparison of the IC_50_ data determined for *P. falciparum* with the reconfirmation data at a fixed 2 μM concentration indicated that mainly compounds with an IC_50_ above 1 μM reconfirmed. For compounds with submicromolar IC_50_ values, all but one compound failed to reconfirm at 2 μM (Table 1). It is possible that due to the higher activity of those compounds, the 2 μM dose might be excessive, resulting in an over-abundance of the inhibitor, which in turn would be more difficult to counteract by CoA supplementation. Hence, the CoA rescue of the six most potent compounds was repeated at a lower compound concentration, namely a value close to their respective IC_80_. At these concentrations, three compounds were rescued to levels of ≥80% and two resulted in approximately 50% rescue (Figure 5A). Only one of the submicromolar active compounds failed to re-confirm at its IC_80_ (Figure 5B). These results confirmed that the activity of a compound can influence its amenability to the CoA rescue, when tested only at one relatively high concentration. It was therefore concluded that future screens should be conducted at two or three distinct concentrations (e.g., at 2 μM, 0.5 μM and 0.1 μM), to be able to reliably identify hits of differing and especially of higher potency levels.

In conclusion, the identification of nine compounds which confirmed with ≥80% CoA rescue is reported (Table 1), four of which showed submicromolar IC_50_ activity (120 nM to 740 nM) and three had IC_50_ values between 1.2 and 1.3 μM against *P. falciparum*. Five of these hit compounds are from the MMV malaria box (MMV665820, MMV000304, Amb2368822 / MMV665980, MMV011438 and MMV000570). Furthermore, three compounds with 50% CoA rescue levels and IC_50_ against *P. falciparum* between 150 nM to 450 nM were identified (Table 1). Importantly, the inhibitors discovered using the novel chemical rescue approach, are not limited to a specific chemical class. Screening for the responsiveness to CoA rescue allowed a broader view to be taken, rather than solely investigating chemically related analogues. As a consequence, this approach yielded a variety of compounds with chemical structures unrelated to pantothenate. With the exeption of MMV011438, all identified compounds have molecular properties compliant with Lipinski’s rule of five (Additional file 1). Additionally, all compounds except STK 668036 have a polar surface area less than 140 ångström squared, a pre-requisite for cell membrane permeation. These favourable chemical properties indicate that some of the compounds identified might be amenable to improvement by medicinal chemistry and therefore could be useful novel starting points for CoA pathway inhibition (Additional file 1).

### Coenzyme A supplementation alleviates effect on parasite growth and cell viability in a dose-dependent manner

**Figure 6:**
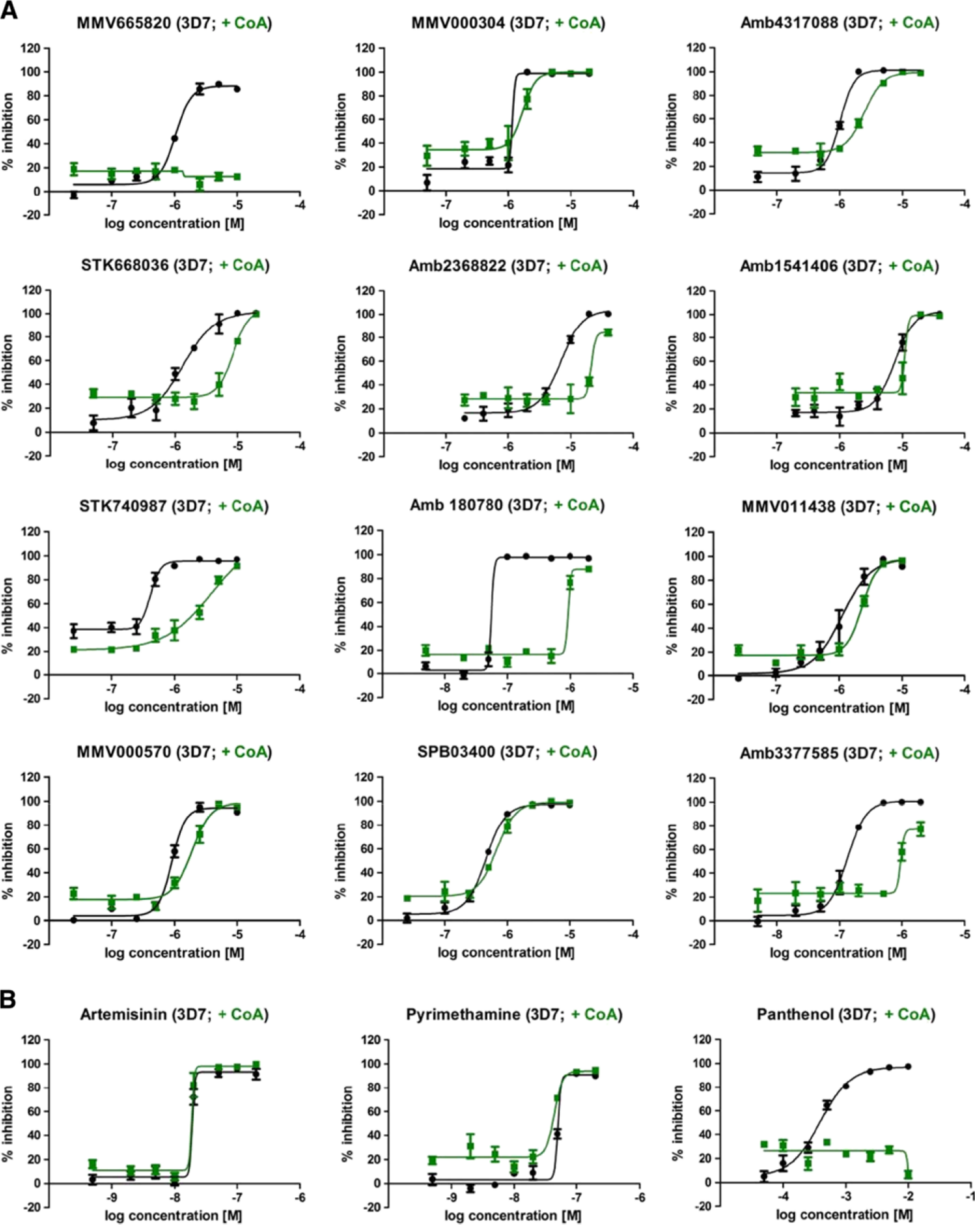
**Coenzyme A supplementation reduces the antiplasmodial effect in a dose-respondent manner. A**: Eight point dose-response curves of hit compounds with (green) and without (black) supplementation of CoA. The optimal CoA concentration for the chemical rescue was determined for each CoA batch and ranged between 0.8-2 mM, once determined the CoA concentration within each experiment was fixed. Generally a shift towards reduced antiplasmodial activity was observed, when CoA was supplemented. Curves represent n = 2 in duplicate point each; error bars indicate +/- standard error of mean (SEM). **B**: Eight point dose-response curves of control compounds with (green) and without (black) supplementation of CoA. The optimal CoA concentration was determined for each CoA batch; once determined the CoA concentration within each experiment was fixed. The activity of the CoA pathway unrelated known anti-malarials artemisinin and pyrimethamine was not reduced by CoA supplementation, whereas the activity of the known CoA pathway inhibitor panthenol was counteracted by CoA addition. Curves represent n = 2 in duplicate point each; error bars indicate +/- standard error of mean (SEM).

The initial screen to identify compounds capable of CoA rescue was performed at a single compound concentration of 2 μM. Subsequently it was observed that compounds with greater antiplasmodial activity had only re-confirmed when tested at lower concentrations more commensurate with their activities on the asexual parasite stages. This observation suggested that the rescue ability might be a dose-dependent effect. To confirm this hypothesis, the CoA rescue ability of the confirmed hits was tested in an eight-point compound dose response in two biological replicates with a fixed amount of CoA. Generally a shift towards reduced antiplasmodial activity was observed, when CoA was supplemented (Figure 6A). For five compounds (MMV665820; STK 668036; STK 740987; Amb180780 and Amb3377585) the observed IC_50_ shift was greater than six times. For four compounds (Amb4317088; Amb2368822/MMV665980; MMV011438 and MMV000570) the IC_50_ value more than doubled upon CoA supplementation and for the remaining three compounds (MMV000304; Amb1541406 and SPB03400) a 1.5 times increase in IC_50_ values was seen. This confirmed that the CoA rescue was a genuine, dose-dependent effect on parasite growth. The impact of CoA addition to the control compounds, artemisinin and pyrimethamine, known anti-malarials that do not affect the CoA pathway, was evaluated. As expected, the activity of these control compounds was not reduced by CoA supplementation, whereas the activity of the known CoA pathway inhibitor panthenol was counteracted by CoA (Figure 6B).

**Figure 7:**
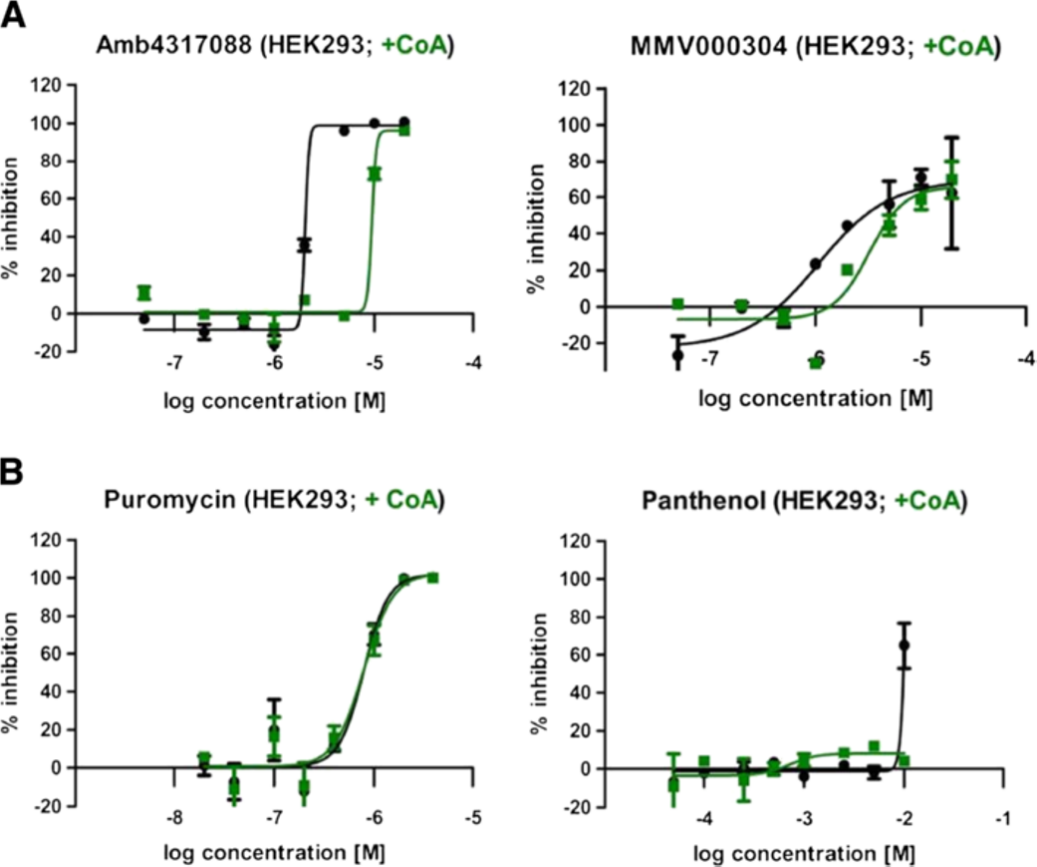
**Coenzyme A supplementation reduces the cytotoxic effect in a dose respondent manner. A**: For both compounds Amb4317088 and MMV000304 the observed cytotoxic effect was reduced upon supplementation of CoA. The optimal CoA concentration was determined for each CoA batch and was fixed within each experiment. Graphs represent n = 2 in duplicate point each; error bars indicate +/- standard error of mean (SEM). **B**: The cytotoxic effect of the control compound puromycin is not reduced by CoA addition, whereas the cytotoxic effect of panthenol is counteracted by CoA. Curves show n = 2 in duplicate point each; error bars indicate +/- standard error of mean (SEM).

Two compounds (Amb4317088 and MMV000304) showed low or no specificity against the malaria parasite over human cells. To determine whether the observed cytotoxicity of these compounds was CoA pathway specific, or a more general growth inhibitory effect, the CoA chemical rescue effect was examined on HEK293 cells to determine whether it could also be observed in a human cell line. For both compounds the cytotoxic effect was reduced upon CoA addition (Figure 7A). As a control, puromycin was used, which has a distinctly different mode of action, namely the inhibition of protein synthesis. CoA addition had no impact on puromycin activity on HEK293 cells (Figure 7B). Panthenol caused a cytotoxic effect on HEK293 cells at 40 μM concentration, and CoA addition was able to completely negate this cytotoxic effect (Figure 7B). These results indicate that compounds Amb4317088 and MMV000304, although not *Plasmodium*-specific, appear to be CoA pathway inhibitors. Presumably these compounds might act on a part of the pathway that is not divergent enough between the parasite and the host to allow for species-specific action. The lack of species specificity indicates that these compounds and their as yet unknown target(s) will not easily be employed for drug development. Nevertheless, these compounds could still serve as valuable tools in the biological investigation of the parasite’s CoA pathway and for interspecies comparisons.

### Parasite stage of action and phenotypic investigation

The parasite viability assay used above is an endpoint assay that spans two development cycles of the parasite. To investigate the parasite stage of action of the identified compounds and the phenotype of compound treated parasites, a light microscopy-based time course study was undertaken. The seven compounds of submicromolar activity were added at time point t0 at their respective IC_80_ concentrations, with and without the addition of 0.8 mM CoA. The 3D7 parasites used in this experiment were age synchronized to within a 2-hr window of the ring stage. Due to assay preparation time, the parasite age at t0 therefore ranged from 0.5 hr to 2.5 hr.

At t0, t18, t24 and t48 parasite cultures for all conditions were centrifuged and Giemsa-stained blood smears were prepared. The 18-hr treatment time point was specifically chosen, since it was expected to see the transition between ring and trophozoite stage in control cultures around this time point (parasite age range 18.5 hr to 20 hr). Therefore any delay in the transition from ring to trophozoite stage under treatment with the compounds was expected to be discernible at this time point. As expected, the DMSO and DMSO + CoA controls showed a stage composition of ~20% rings and ~80% young trophozoites at t18, then progressed to middle aged trophozoites at t24 and further to ~90% ring stages at t48 (Additional file 2A).

**Figure 8:**
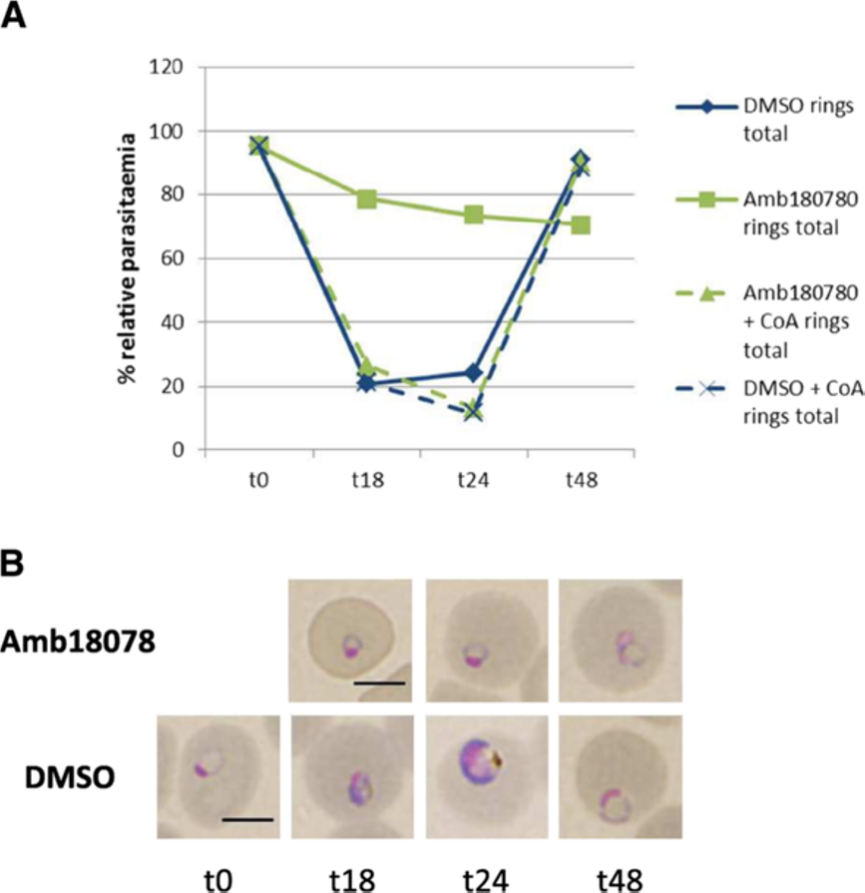
**Exemplary time course (compound Amb180780) for parasite stage of action and phenotypic investigation. A**: Parasites treated with Amb180780 (solid green line) appear to be arrested at the ring stage throughout the time course. CoA addition rescues the progression through the parasite stages (dashed green line); graphs show n = 1, the Giemsa smear was taken from 16 pooled replicate wells. A minimum of 100 parasites were phenotypically assessed for each time point. Relative parasitaemia values are shown. **B**: Representative Giemsa stained bright field microscopic images showing the predominant parasite phenotype at each time point for compound treatment (top panel) and DMSO control (bottom panel). Scale bar 5 μm.

Compound ***Amb180780*:** Less than 13% of the compound-treated parasites were able to progress from the ring to the trophozoite stage. The parasite appears to be arrested at a small ring stage throughout the time course (Figure 8). Dead parasite counts increase steeply up to 28.6% relative parasitaemia at t48. Whilst CoA addition could not prevent the increased death rate of 20% at t24, at t48 the death rate in the CoA supplemented culture drops again to control levels (Additional file 2B). Overall, the relative parasite numbers and progression through the stages was rescued very well by CoA addition. In conclusion, compound Amb180780 would appear to arrest parasite development at the ring stage.

Compound ***MMV665820*:** Parasites developed normally from rings into young trophozoites around t18. At t24 the relative parasitaemia of trophozoites showing abnormal morphology increased to 42.9%, which equals approximately two-thirds of all counted living trophozoites at this time point. The main morphological change observed was an irregular parasite outline, resulting in a ‘spiky’ appearance resembling a hedgehog (Additional file 2C). At t48 there were almost no rings visible, indicating that the normal transition was not occurring. The relative count of abnormal trophozoites decreased to 26.6% (approximately one-third of living trophozoites), but the relative parasitaemia of dead or dying trophozoites increased to 13.8% (Additional file 2C). Together these findings indicate that the main compound action is on the trophozoite stage, which is affected morphologically or killed before it can further progress in its development. CoA addition effectively rescued both parasite development and morphology throughout the time course studied (Additional file 2C).

#### STK 740987

For this compound, no change in the stage composition of rings and trophozoites compared to the controls was noted throughout the time course. The only difference observed was an increase in dead or dying parasites at t24 to around 16%, compared to around 8% in the controls. However this difference was no longer apparent at t48 (Additional file 2D).

#### MMV000570

A significant delay in the development time of the compound-treated parasites was observed at IC_80_ concentrations. Parasites were mostly in ring form at t24 and developed into trophozoites only by t48, when untreated parasites had already completed their development cycle and were predominantly in the ring form again. Addition of CoA did not alleviate this developmental delay (Additional file 2E). However, at 24 and 48 hr an increase in dead or dying parasites was observed in compound treated samples to around 28 and 20%, respectively, which was reduced to 13.4 and 8.3%, respectively by CoA supplementation. These data suggest that MMV000570 affects both the development of the ring and trophozoite stage of the parasite.

#### MMV011438

The development from rings into trophozoites was delayed for both the compound-treated and compound plus CoA-treated samples, resulting in a higher relative ring and lower relative trophozoite parasitaemia at t18 compared to the DMSO control (Additional file 2F). From t24 a steep rise of trophozoites with abnormal morphology were observed in the compound-treated sample. The morphological changes seen were most pronounced at t48 in mature trophozoites, which showed vacuoles in the cytoplasm and a ‘clumpy’ appearance of their DNA. This abnormal morphology affected 58% of all counted living trophozoites at t48 (33.9% relative parasitaemia). Additionally a high number (24.8% relative parasitaemia) of schizonts with abnormal morphology was also observed at t48 (Additional file 2F). The schizonts showed an irregular outline and ‘clumpy’ DNA. Almost no rings were observed at t48. In summary, these findings suggest that MMV011438 slows parasite development and mainly affects the transition from mature trophozoite to schizont. Progression to the ring stage was greatly delayed or even inhibited. CoA addition successfully rescued the parasites with respect to correcting the morphological changes in both trophozoites and schizonts and the parasites progressed normally to produce rings at t48.

#### SPB03400

No change in the relative parasitaemia of rings or trophozoites compared to the untreated controls was observed for this compound throughout the time course. The only difference observed was an increase in ring form parasites showing abnormal morphologies at t48 to around 50%, compared to around 16-27% in the controls (Additional file 2G).

#### Amb3377585

A slight developmental delay from ring form to trophozoites was observed for both the compound-treated and compound plus CoA-treated samples, resulting in an approximately two times higher relative ring and approximately half the relative trophozoite parasitaemia at t18 compared to the DMSO control (Additional file 2H). From t24 onwards an increased death rate of parasites in the compound treated sample to 49% of relative parasitaemia was observed, compared to 7-11% in controls. At t48 almost no rings developed during compound treatment and a dramatic increase of trophozoites exhibiting abnormal morphology were reported, with abnormal trophozoites making up 30.5% of relative parasitaemia and 51% of all counted living trophozoites (Additional file 2H). The morphological alterations displayed by the trophozoites resulted in a large variety of phenotypes. In summary, the data indicate that the main compound action is in killing both the ring and the trophozoite stage of the parasite from approximately t24. Additionally compound treatment causes a multitude of abnormal phenotypic changes in trophozoites, which hinder further progress in parasite development to the ring stage at t48. Apart from the initial slight delay in development at t18, a very good rescue of parasite development, death rates and morphology throughout the time course was demonstrated upon addition of CoA (Additional file 2H).

## Discussion

The CoA synthesis pathway is essential for *P. falciparum* survival. Despite the conservation of the chemical synthesis steps among eukaryotes, the enzymes involved are not highly conserved between species, which renders them promising targets for chemotherapy. Ever since the essentiality of the CoA pathway for parasite survival was demonstrated, the main medicinal chemistry focus in this area has been on the chemical modification of pantothenate, the first substrate in the synthesis pathway [10, 21]. Despite the improvements that have been achieved, none of the pantothenate analogues investigated to date provided lead compounds suitable for drug development. Therefore the objective was to find a new approach for the discovery of novel, chemically diverse inhibitors of *P. falciparum* proliferation, that act on the parasite CoA synthesis pathway. To this aim an established *P. falciparum* imaging assay was modified by the addition of a chemical rescue step to screen for inhibitors of the CoA pathway. As a proof of principle it was shown that the chemical rescue approach, namely the supplementation of the pathway’s end product, CoA, could negate the growth inhibitory effect of the known CoA synthesis inhibitor panthenol.

Then the chemical rescue method was used to screen a set of 144 prioritized compounds as well as the compounds of the MMV malaria box (400) to identify new *P. falciparum* inhibitors that interfere with the parasite’s CoA synthesis. Six compounds that were fully amenable to chemical rescue were identified and one compound which was rescued by greater than 50% of the control values at 2 μM compound concentration. Three hits had IC_50_ values against *P. falciparum* between 1.2-1.3 μM and two with submicromolar potency of 580 nM and 380 nM, respectively. Furthermore it was shown that the CoA chemical rescue is a dose-dependent effect and that out of compounds with higher antiplasmodial activity, i.e., IC_50_ ranging between 120 to 740 nM, three could be rescued to ≥80% of control values when tested close to their individual IC_80_ values. Two more could be rescued to approximately 50% and only one did not confirm at its IC_80_.

When the inhibitors were tested in dose response with and without CoA addition, a concentration dependent shift towards reduced compound activity was observed, when CoA was supplemented. Furthermore, the cytotoxicity observed for two compounds against human HEK293 cells could also be reduced by CoA addition. This implies that the chemical rescue principle can also be applied in mammalian cell culture. The reduction in cytotoxicity after CoA supplementation suggests that these compounds are not randomly toxic but might act on parts of the CoA pathway that are not divergent enough between *Plasmodium* and human to allow for species specificity.

Phenotypic time course investigations revealed a variety of different actions by the compounds, including developmental delays and morphological changes, as well as killing the parasite. Amb180780 would appear to arrest parasite development at the ring stage, which is rescued effectively by CoA addition. The main action of compound MMV665820 is on the trophozoite stage, causing morphological changes and killing the parasite, thus preventing further progression to the ring stage. Supplementation of CoA provides good rescue of both morphological changes and death rates. MMV011438 slows parasite development and causes severe morphological changes, especially in mature trophozoites and schizonts, both of which can be counteracted by CoA addition. MMV000570 affects both the development of rings and trophozoites and it is the only compound for which addition of CoA was not found to rescue the observed development delay, however CoA addition was able to reduce the number of dead and dying as well as morphologically altered parasites. Amb3377585 was found to kill both the ring and the trophozoite stage of the parasite, additionally causing a variety of phenotypic changes in trophozoites. Apart from a slight initial development delay, CoA provides rescue of parasite development, death rates and morphology. For the compounds STK 740987 and SPB03400 no obvious changes in parasite development compared to controls were observed at the investigated time points. This wide variety of discernible compound actions is very promising, since it indicates that the compounds might act through diverse mechanisms of action and/or on different targets within the CoA pathway. This opens the possibility that some of the compounds might show synergistic effects when used in combination, e.g., between Amb180780, which arrests the parasite in the ring stage and any of the compounds that were shown to kill the parasite at the ring stage. Such compound combinations could improve the antiplasmodial potency further, whilst still acting specifically on the parasite’s CoA pathway.

Importantly, the described novel approach yielded compounds with proven antiplasmodial activity for a variety of different chemotypes. This opens up the possibility of completely new avenues for medicinal chemistry improvement. Furthermore, the described approach has been established in 384-well format and is suitable for use in HTS of large compound libraries. However, the high cost of CoA is at the moment prohibitive. Therefore in this pilot screen a collection of only 500 prioritized compounds was investigated, including those of the MMV malaria box. To overcome the limitations imposed by the costs associated with CoA, it may be possible to synthesize CoA in-house or to initiate custom synthesis in larger quantities. However, the batch specific toxicity issues observed in the purchased CoA already hint towards inherent challenges that would need to be overcome when embarking on large-scale synthesis of CoA.

The herein-identified inhibitors can readily be used as tools for further molecular and functional investigation of the CoA synthesis pathway. The two inhibitors that were identified not to be *Plasmodium*-specific might prove useful for interspecies comparisons. The cytotoxic effect observed on the human cell line HEK293 for two compounds was also amenable to chemical rescue by CoA supplementation, which implies the suitability for this method in mammalian cell culture. Furthermore, the approach might provide useful in the investigation of other parasite species, especially those in which molecular manipulation techniques are still in their infancy, therefore precluding the use of genetic approaches such as gene knockdown and over-expression as investigation tools. Since molecular manipulation of *P. falciparum* is still complex and time consuming, the chemical rescue concept could also be a useful tool for the investigation of other plasmodial pathways, as recently published by Bowman *et al.* for compounds affecting the apicoplast [22]. A key issue is that the molecule intended for any such rescue approach must be able to pass through both the erythrocytic and the parasite membranes. When relying on the uptake of a compound from the culture media as in the present study, it is to be expected that high extracellular concentrations of the rescue compound might be needed. This firstly must be tolerated by the parasite without in itself causing toxicity and secondly it raises the experimental costs considerably. Therefore investigations into facilitating targeted delivery might greatly enhance the potential of the chemical rescue technique.

## Conclusions

Herein it is demonstrated how a chemical rescue concept can be used in 384-well format to screen for inhibitors of a vital synthesis pathway in *P. falciparum*. By focussing on the end result (rescue of growth inhibition), rather than a specific target molecule, this approach allows the identification of a multitude of structurally different inhibitors that interfere with the parasite’s CoA synthesis pathway. A set of 12 chemically diverse *P. falciparum* inhibitors amenable to the CoA chemical rescue were identified. The compound IC_50_ values ranged between 120 nM and 6 μM. Phenotypic observations were made over a time course for seven submicromolar active compounds, which revealed that the compounds act in different ways to prevent parasite growth and parasite stage development. Further molecular and functional investigations to identify the synergistic potential, as well as the molecular targets and mechanisms of action of the newly identified inhibitors are in progress. The hit compounds identified are chemically diverse offering valuable new starting points for medicinal chemistry and tools for target investigation. The screening method described can be applied to the investigation of medium-sized prioritized compound libraries. The method yields hit compounds with demonstrated antiplasmodial action and defined species selectivity as well as being a first step in determining the mode of action of novel anti-malarial compounds, namely inhibition of the CoA synthesis pathway. Collectively, the CoA rescue approach is proposed as a novel valuable tool for future anti-malarial drug discovery.

## Electronic supplementary material

Additional file 1: **Chemical properties of confirmed coenzyme A rescue compounds.** (DOCX 81 KB)

Additional file 2: **Time course of parasite stage of action and treatment phenotype.** Graphs show n = 1, Giemsa smears taken from 16 replicate wells. A minimum of 100 parasites were phenotypically assessed for each time point. Values are given as relative parasitaemia. Scale bars indicate 5 μm. **A:** Top: ring form (blue) and trophozoites (red) over 48 hr in control cultures +/- CoA. Bottom: predominant phenotype at each time point. **B:** Death rate increase with Amb180780 (orange, solid); rescued at t48 by CoA (dashed). **C:** Top graph / images: MMV665820 (green, solid) prevents ring formation at t48; rescued by CoA (dashed). Middle graph: Increased abnormal morphologies (red, solid); prevented by CoA (dashed). Bottom graph: Increased trophozoite death rate (orange, solid), counteracted by CoA (dashed). **D:** Top: No stage composition change with STK 740987. Bottom: Increased death rate at t24 (orange, solid); rescued by CoA (dashed). **E:** Top graph / images: CoA non-responsive development delay of MMV000570; development into trophozoites only by t48 (green, solid and dashed). Bottom graph / images: CoA decreased number of dead parasites (orange, dashed) and rescued trophozoite morphology at t48. **F:** Top graph / images: MMV011438 treatment prevented development into ring forms at t48 (orange, solid). Initial development delay at t24 with CoA, but ring formation normal at t48 (dashed). Bottom graph / images: trophozoites and schizonts with abnormal morphology (red, solid), rescued by CoA addition (dashed) **G:** Top: Stage composition unchanged with SPB03400. Bottom: increased abnormal ring morphologies at t48 compared to controls (solid red line). **H:** Top graph / images: Amb3377585 treatment prevented development into ring forms (green, solid); Initial development delay at t18 with CoA, but ring formation normal at t48 (dashed). Middle graph: increased death rate with treatment (orange, solid); prevented by CoA (dashed). Bottom graph / images: morphologically altered trophozoites at t48 (red, solid); rescued by CoA (dashed). (PPTX 515 KB)

## Abbreviations

CoA: Coenzyme A
HEK293: Human embryonic kidney cell line
HTS: High throughput screening
MMV: Medicines for malaria venture.
