# Supplementary material for: A novel approach for the discovery of chemically diverse anti-malarial compounds targeting the Plasmodium falciparum Coenzyme A synthesis pathway
Source: Malar J. 2014 Aug 31;13:343. doi: 10.1186/1475-2875-13-343 (PMC4168161; doi:10.1186/1475-2875-13-343)
Supplement: Supplementary file 1 — Additional file 1: Chemical properties of confirmed coenzyme A rescue compounds. (DOCX 81 KB) [file 12936_2014_3390_MOESM1_ESM.docx]

|  |  |  | **Lipinski and related properties** | | | | | | |  |
| --- | --- | --- | --- | --- | --- | --- | --- | --- | --- | --- |
| **compound ID** | **Structure** | **CAS registry number** | **FRB** | **HBA** | **HBD** | **logP (25 °C)** | **MW** | **Number of violations** | **Lipinski compliant** | **PSA**  **[Å^2^]** |
| MMV665820 / 5534045 |  | 255843-62-0 | 1 | 3 | 0 | 1.942±0.970 | 293.53 | 0 | yes | 35.5 |
| MMV000304 / Amb636705 |  | 439148-15-9 | 4 | 3 | 1 | 5.128±0.806 | 314.38 | 1 | yes | 34.2 |
| Amb4317088 |  | 17579-85-0 | 2 | 3 | 0 | 3.605±0.734 | 276.29 | 0 | yes | 51.2 |
| STK 668036 |  | 183876-38-2 | 0 | 20 | 0 | -0.142±1.693 | 384.19 | 1 | yes | 255 |
| Amb2368822 / MMV665980 |  | 294877-78-4 | 4 | 3 | 3 | 3.606±0.322 | 332.5 | 1 | yes | 76.4 |
| Amb1541406 |  | 379253-38-0 | 4 | 3 | 0 | 5.103±0.571 | 294.39 | 1 | yes | 27.1 |
| STK 740987 |  | 5505-85-1 | 4 | 5 | 0 | 2.720±0.489 | 319.38 | 0 | yes | 81.2 |
| Amb180780 |  | 923915-34-8 | 2 | 3 | 0 | 2.482±1.017 | 265.38 | 0 | yes | 120 |
| MMV011438 / STK039514 |  | 543696-46-4 | 6 | 6 | 1 | 6.022±0.883 | 546.59 | 4 | No | 79.2 |
| MMV000570 / C614-0191 |  | 931719-15-2 | 1 | 4 | 0 | 2.376±0.762 | 263.25 | 0 | yes | 45.2 |
| SPB03400 |  | 263410-85-1 | 4 | 7 | 0 | 1.594±0.553 | 280.3 | 1 | yes | 119 |
| Amb3377585 |  | 312505-78-5 | 1 | 4 | 0 | 2.376±0.762 | 263.25 | 0 | yes | 60.2 |

All values of chemical properties were retrieved using SciFinder. **FRB:** freely rotatable bonds; **HBA:** hydrogen bond donor; **HBA:** hydrogen bond acceptor; **logP:** partition coefficient; **MW:** molecular weight; **PSA:** polar surface area.
