# Supplementary material for: A novel approach for the discovery of chemically diverse anti-malarial compounds targeting the Plasmodium falciparum Coenzyme A synthesis pathway
Source: Malar J. 2014 Aug 31;13:343. doi: 10.1186/1475-2875-13-343 (PMC4168161; doi:10.1186/1475-2875-13-343)
Supplement: Supplementary file 2 — Additional file 2: Time course of parasite stage of action and treatment phenotype. Graphs show n = 1, Giemsa smears taken from 16 replicate wells. A minimum of 100 parasites were phenotypically assessed for each time point. Values are given as relative parasitaemia. Scale bars indicate 5 μm. A: Top: ring form (blue) and trophozoites (red) over 48 hr in control cultures +/- CoA. Bottom: predominant phenotype at each time point. B: Death rate increase with Amb180780 (orange, solid); rescued at t48 by CoA (dashed). C: Top graph / images: MMV665820 (green, solid) prevents ring formation at t48; rescued by CoA (dashed). Middle graph: Increased abnormal morphologies (red, solid); prevented by CoA (dashed). Bottom graph: Increased trophozoite death rate (orange, solid), counteracted by CoA (dashed). D: Top: No stage composition change with STK 740987. Bottom: Increased death rate at t24 (orange, solid); rescued by CoA (dashed). E: Top graph / images: CoA non-responsive development delay of MMV000570; development into trophozoites only by t48 (green, solid and dashed). Bottom graph / images: CoA decreased number of dead parasites (orange, dashed) and rescued trophozoite morphology at t48. F: Top graph / images: MMV011438 treatment prevented development into ring forms at t48 (orange, solid). Initial development delay at t24 with CoA, but ring formation normal at t48 (dashed). Bottom graph / images: trophozoites and schizonts with abnormal morphology (red, solid), rescued by CoA addition (dashed) G: Top: Stage composition unchanged with SPB03400. Bottom: increased abnormal ring morphologies at t48 compared to controls (solid red line). H: Top graph / images: Amb3377585 treatment prevented development into ring forms (green, solid); Initial development delay at t18 with CoA, but ring formation normal at t48 (dashed). Middle graph: increased death rate with treatment (orange, solid); prevented by CoA (dashed). Bottom graph / images: morphologically altered trophozoites at t48 [file 12936_2014_3390_MOESM2_ESM.pptx]

## Slide 1
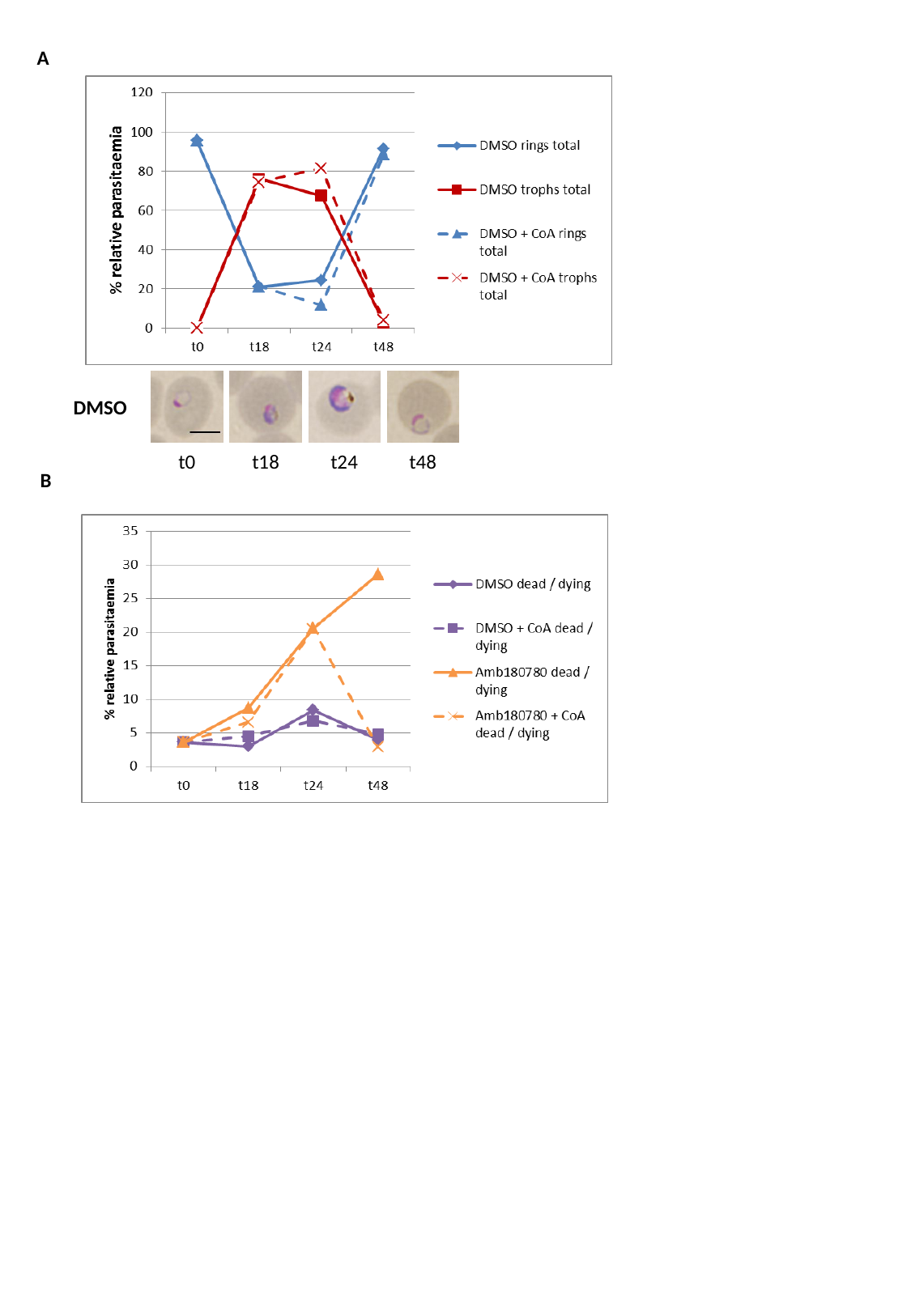

A
DMSO
t0
t18
t24
t48
B

## Slide 2
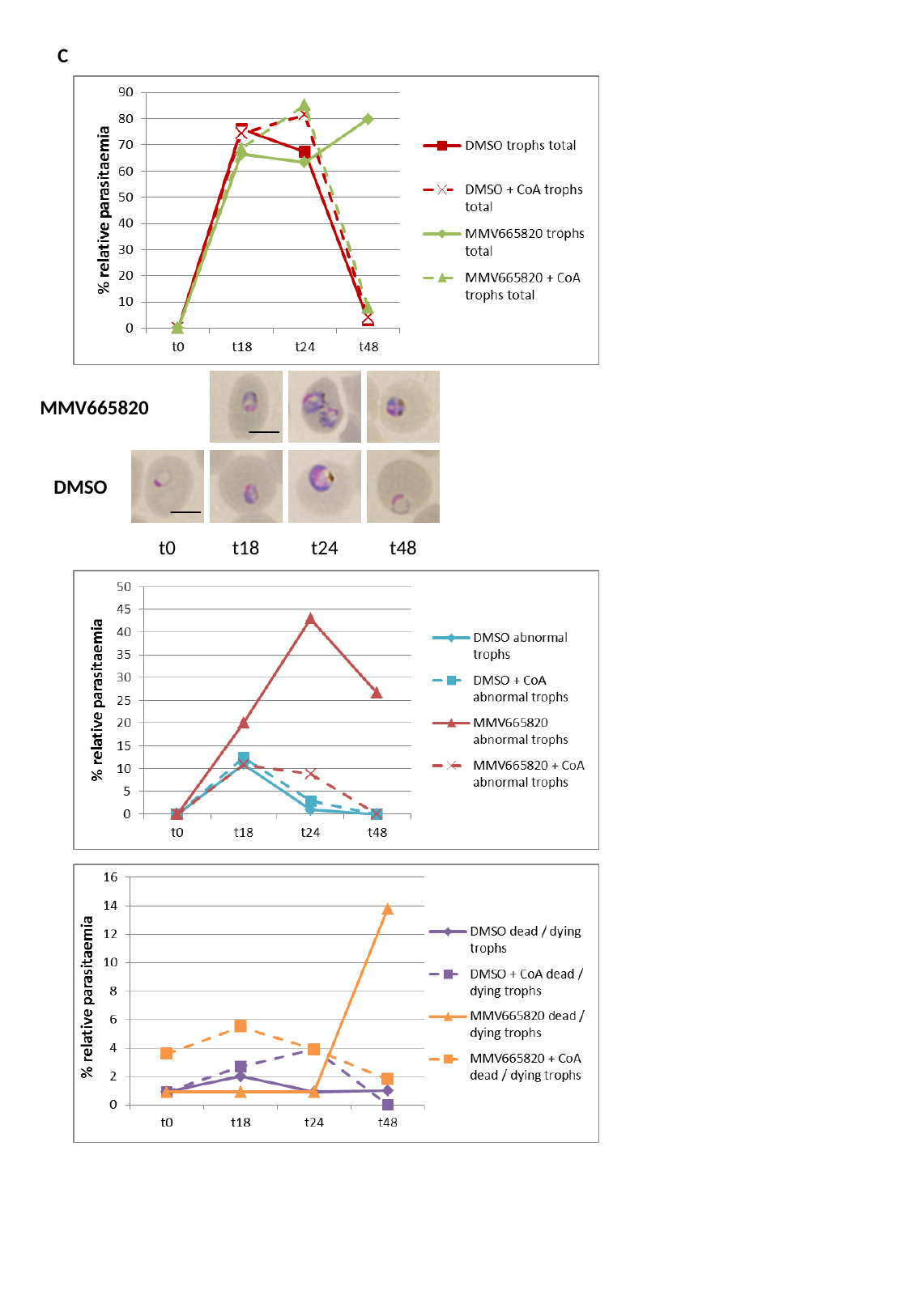

C
MMV665820
DMSO
t0
t18
t24
t48

## Slide 3
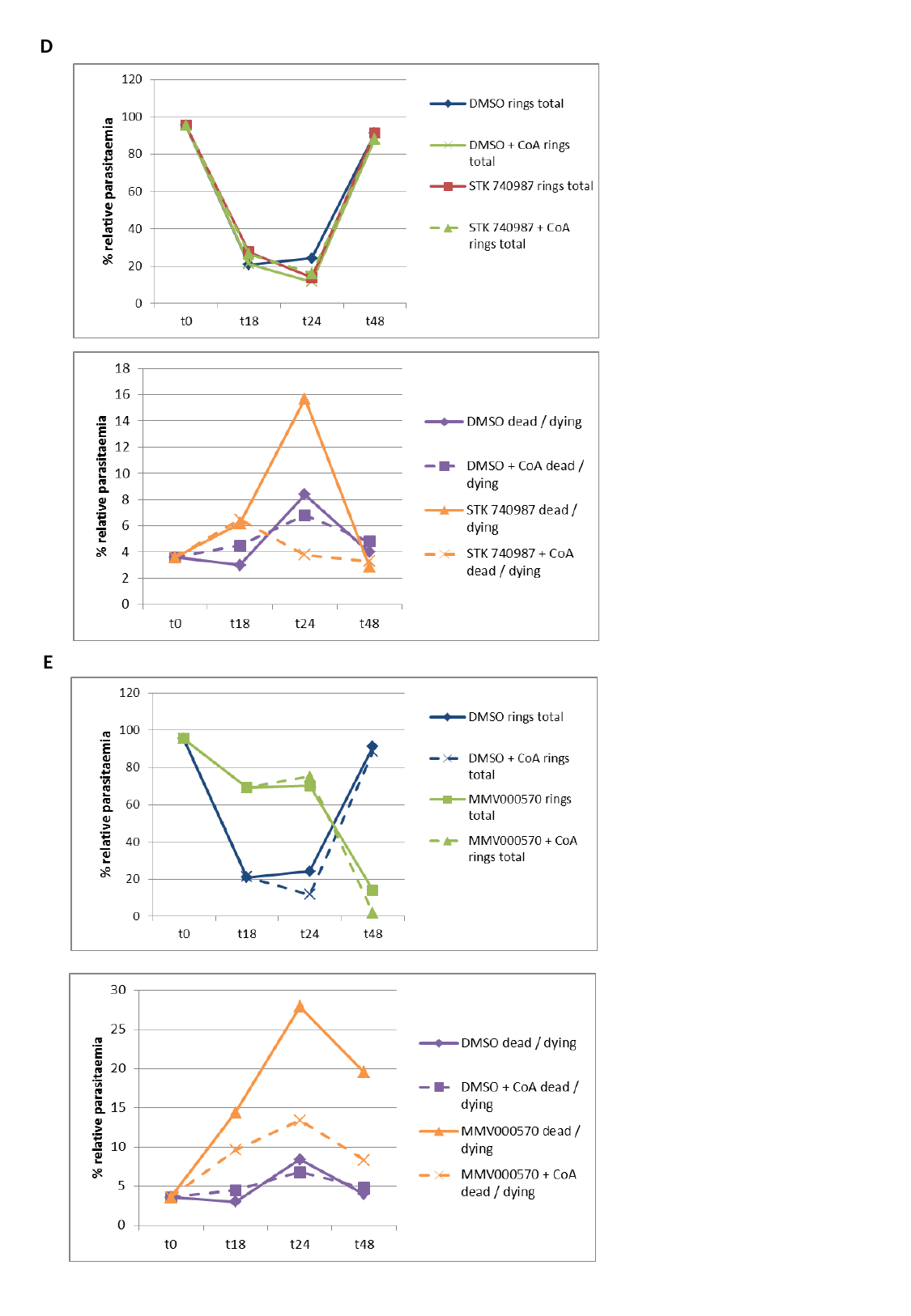

D
E

## Slide 4
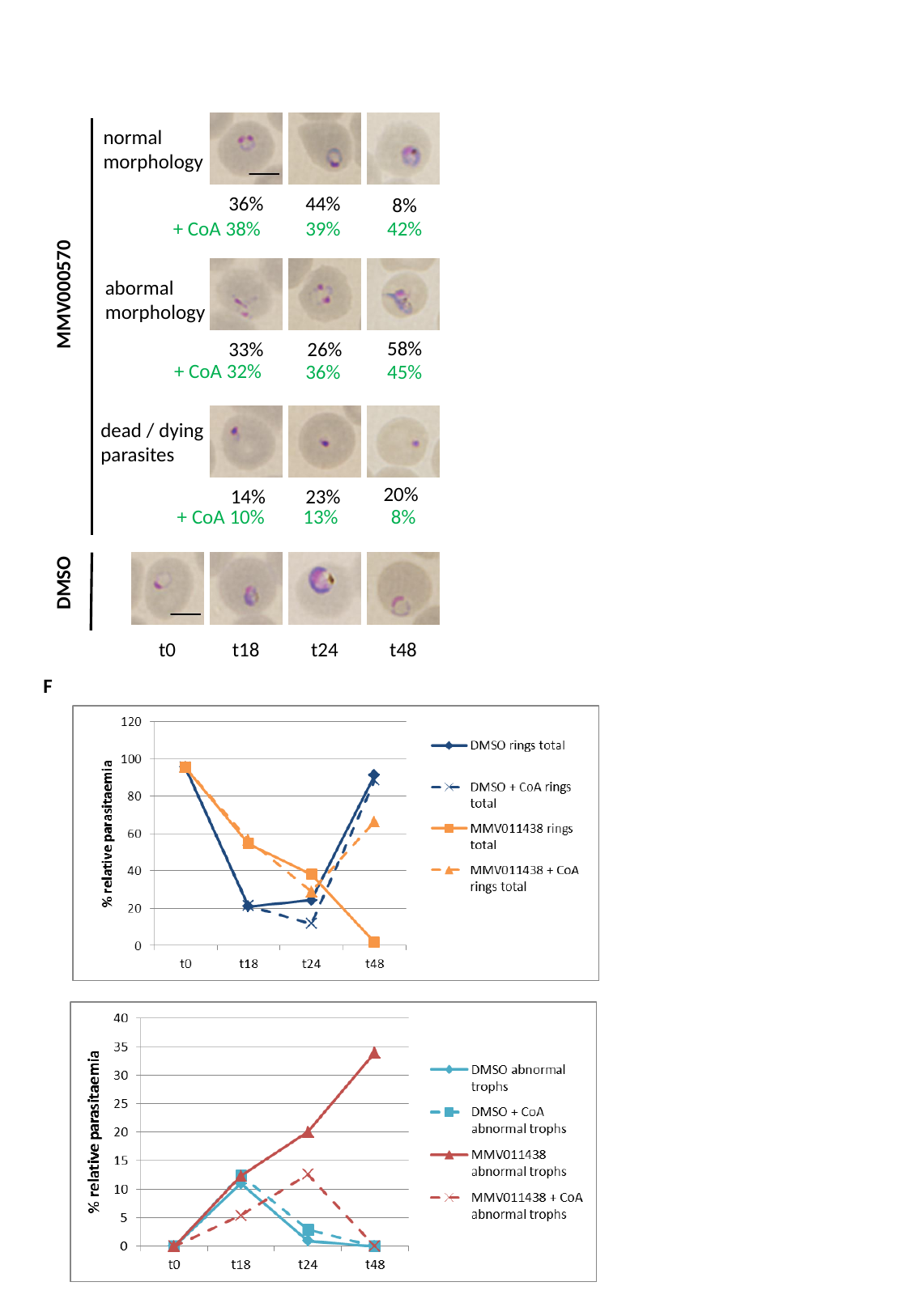

normal
morphology
44%
36%
8%
42%
39%
+ CoA 38%
MMV000570
abormal
morphology
58%
26%
33%
+ CoA 32%
36%
45%
dead / dying
parasites
20%
14%
23%
+ CoA 10%
13%
8%
DMSO
t0
t18
t24
t48
F

## Slide 5
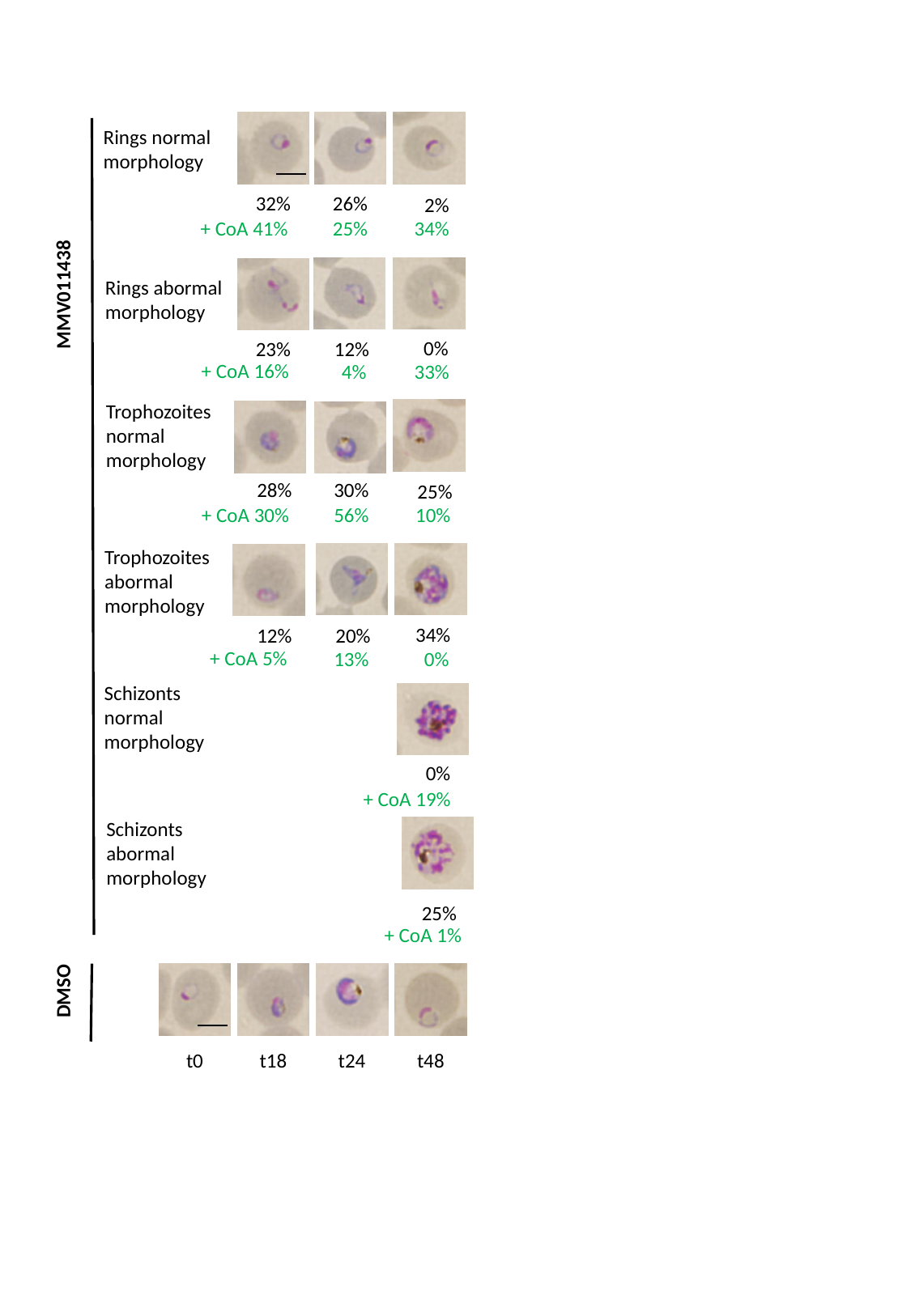

Rings normal
morphology
26%
32%
2%
34%
25%
+ CoA 41%
MMV011438
Rings abormal
morphology
0%
12%
23%
+ CoA 16%
4%
33%
Trophozoites
normal
morphology
30%
28%
25%
10%
56%
+ CoA 30%
Trophozoites
abormal
morphology
34%
20%
12%
+ CoA 5%
13%
0%
Schizonts
normal
morphology
0%
+ CoA 19%
Schizonts
abormal
morphology
25%
+ CoA 1%
DMSO
t0
t18
t24
t48

## Slide 6
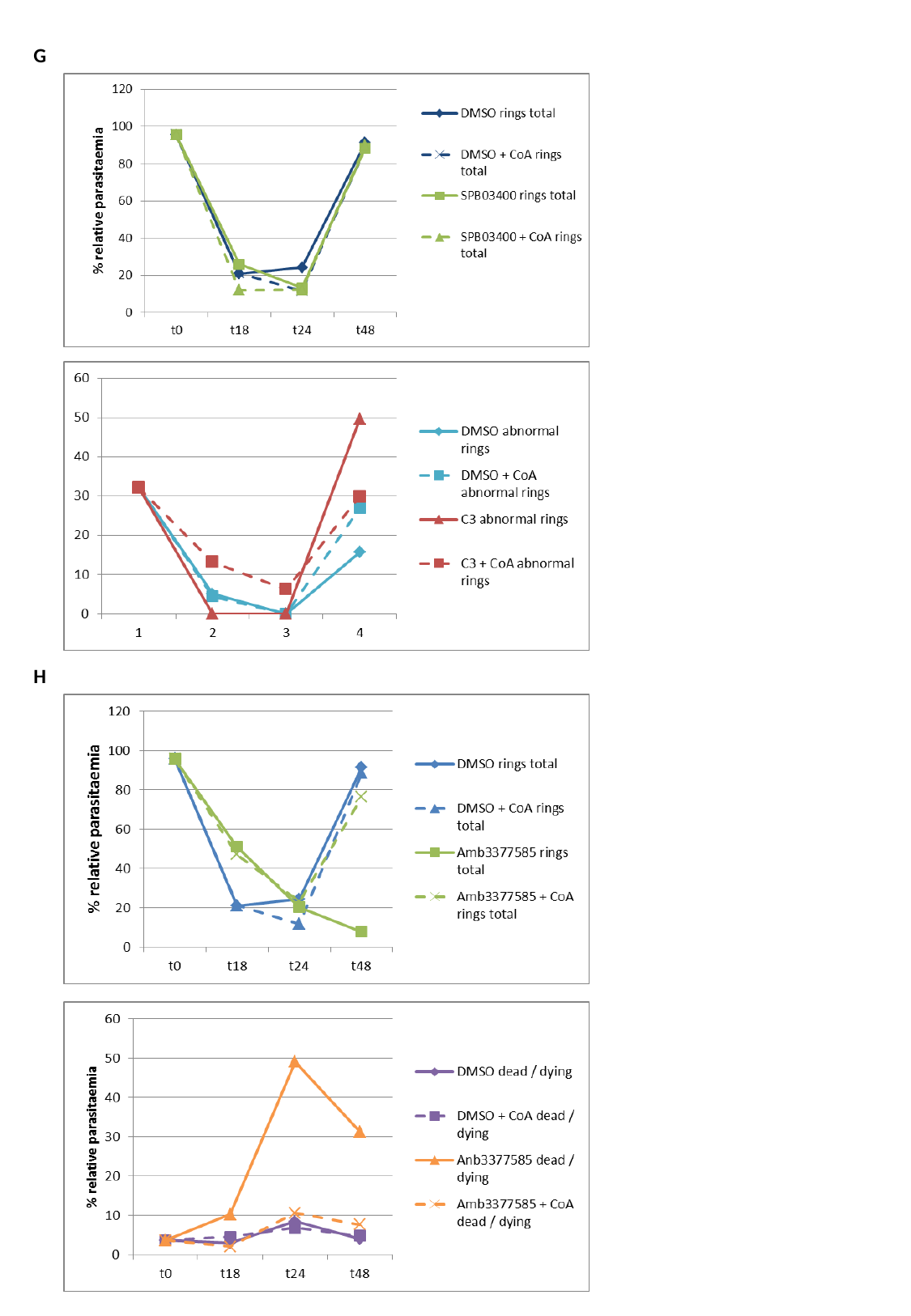

G
H

## Slide 7
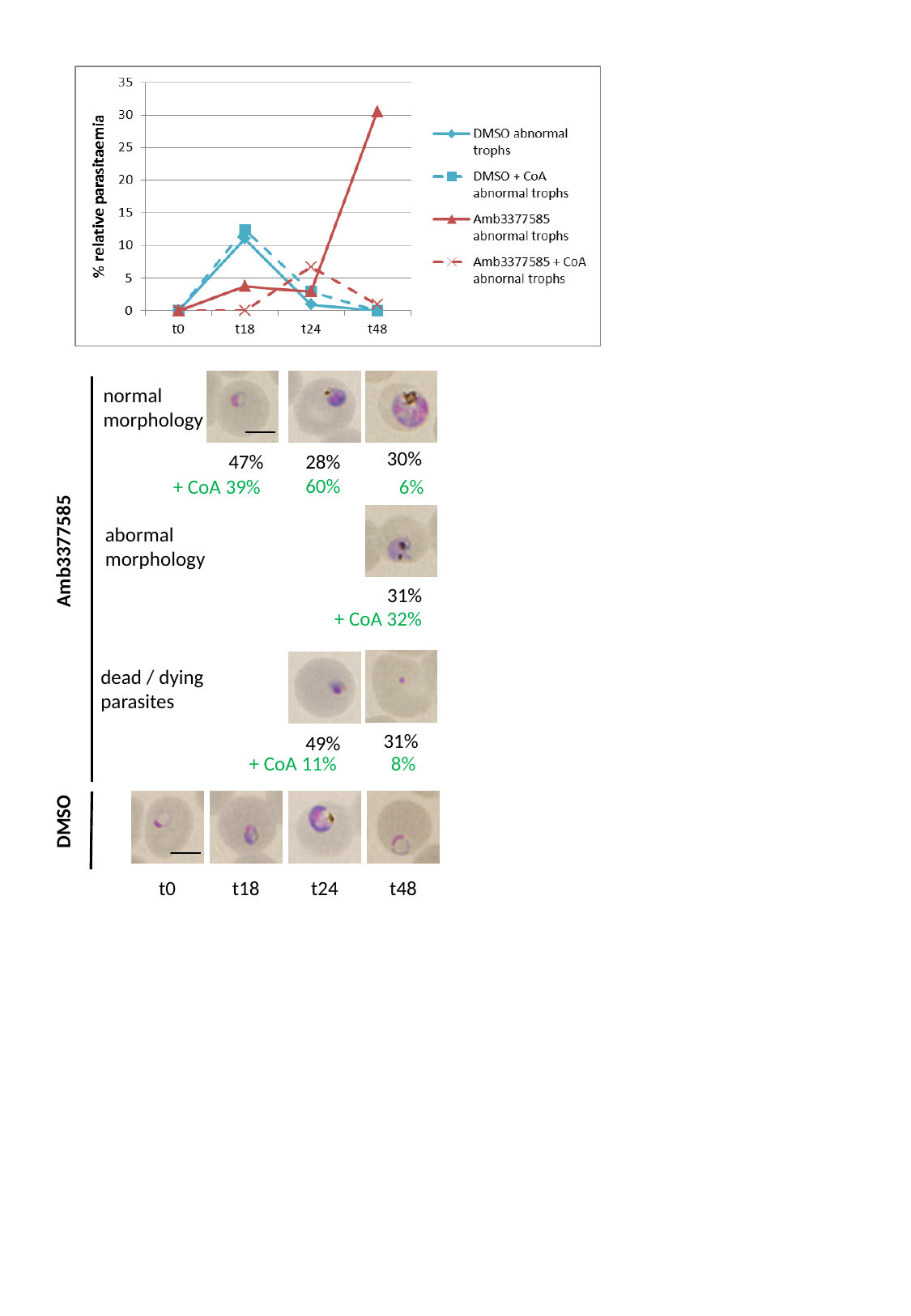

normal
morphology
30%
28%
47%
60%
+ CoA 39%
6%
Amb3377585
abormal
morphology
31%
+ CoA 32%
dead / dying
parasites
31%
49%
8%
+ CoA 11%
DMSO
t0
t18
t24
t48
